# Who is protected? Determinants of hepatitis B infant vaccination completion among a prospective cohort of migrant workers in Thailand during the COVID-19 pandemic

**DOI:** 10.1186/s12939-022-01802-5

**Published:** 2022-12-30

**Authors:** Mary Ellen Gilder, Chanapat Pateekhum, Ahmar Hashmi, Chanchanok Aramrat, Ko Ko Aung, Wimon Miket, Cindy S. Chu, December Win, Marieke Bierhoff, Wichuda Wiwattanacharoen, Wichuda Jiraporncharoen, Chaisiri Angkurawaranon, Rose McGready

**Affiliations:** 1 Department of Family Medicine, Faculty of Medicine, Chiang Mai University, 110 Intawaroros Road, Sriphum, Muang, Chiang Mai, 50200 Thailand; 2 Institute for Implementation Science, University of Texas Health Sciences Center (UTHealth), Houston, USA; 3 Department of Health Promotion and Behavioral Sciences, School of Public Health, University of Texas Health Sciences Center (UTHealth), Houston, USA; 4 Shoklo Malaria Research Unit, Mahidol-Oxford Tropical Medicine Research Unit, Faculty of Tropical Medicine, Mahidol University, Mae Sot, Tak, Thailand; 5 Centre for Tropical Medicine and Global Health, Nuffield Department of Medicine, University of Oxford, Oxford, UK; 6 Sarapee Hospital, Sarapee, Chiang Mai, Thailand

**Keywords:** EPI, Myanmar, Immunization, Health services, Documentation, Tak, Mae Sot, Chiang Mai, Migration

## Abstract

**Background:**

Hepatitis B causes significant disease and death globally, despite the availability of effective vaccination. Migration likewise affects hundreds of millions of people annually, many of whom are women and children, and increases risks for poor vaccine completion and mother to child transmission of hepatitis B. In the neighbouring countries of Thailand and Myanmar, vaccine campaigns have made progress but little is known about the reach of these programs into migrant worker communities from Myanmar living in Thailand.

**Methods:**

A cohort of 253 postpartum women (53 urban migrants in Chiang Mai and 200 rural migrants in Tak Province) were surveyed about their Hepatitis B knowledge and willingness to vaccinate their children between September 10, 2019 and March 30, 2019. They were subsequently followed to determine vaccine completion. When records of vaccination were unavailable at the birth facility, or visits were late, families were contacted and interviewed about vaccination elsewhere, and reasons for late or missed vaccines.

**Results:**

Though women in Tak province displayed better knowledge of Hepatitis B and equal intention to vaccinate, they were 14 times less likely to complete Hepatitis B vaccination for their children compared to migrants in Chiang Mai. Tak women were largely undocumented, had private (non-profit) insurance and had more transient residence. In Chiang Mai migrant women were mostly documented and had full access to the Thai national health services. Though minor individual and facility-level differences may have contributed, the major driver of the disparity seems to be the place of migrants within local socio-political-economic systems. The COVID-19 pandemic further disproportionately affected Tak province migrants who faced severe travel restrictions hampering vaccination. Sixty percent of families who were lost to vaccine follow-up in Tak province could not be contacted by phone or home visit. Chiang Mai migrants, with 86.8% vaccine completion, nearly reached the target of 90%.

**Conclusions:**

Achievement of high levels of hepatitis B vaccination in migrant communities is important and feasible, and requires inclusive policies that integrate migrants into national health and social services. This is more urgent than ever during the COVID-19 era.

**Supplementary Information:**

The online version contains supplementary material available at 10.1186/s12939-022-01802-5.

## Background

In 2019 almost 300 million individuals were chronically infected with hepatitis B, and over 800,000 people died from liver damage due to hepatitis B infection [1]. Mother-to-child transmission at birth is responsible for the majority of chronic infections and adverse outcomes because there is a high risk of persistent infection when exposure occurs in the neonatal period [2]. Universal childhood vaccination campaigns initiated in the 1990s have been effective in reducing mother-to-child transmission [3]. However, these campaigns require 90% coverage to reduce infections to < 1% [4], a goal that is hard to reach in many low-resource settings, especially in rural [5] and marginalized migrant populations [6, 7]. The COVID-19 pandemic has disrupted routine health services globally, resulting in decreased vaccination rates affecting millions of children [8], but differential effects on marginalized populations have not been explored.

Migration has been a major component of the global social, political, economic, and health landscape of the past century and this trend is expected to continue [9]. Migrants, especially those with inadequate legal documentation, are particularly vulnerable to disease and injury [10], and there is an urgent need for a greater understanding of the determinants of migrant health to inform effective and equitable public health strategies [11]. Both preventative and curative healthcare shortfalls for migrants and refugees have been exacerbated during the COVID-19 pandemic [12].

There are over 2 million officially registered migrant workers in Thailand, the majority of whom come from Myanmar, and an unknown number of undocumented migrants. In Tak province, Thailand, which shares a porous border with Kayin state, Myanmar, the estimated 200,000 migrant workers are largely undocumented, crossing the border by irregular channels and working for approximately half of the legal minimum wage [13–15]. Migrant workers elsewhere in Thailand, including Chiang Mai, are more likely to be documented and receive minimum wage salaries [14]. Legal documentation for migrant workers often provides access to Thailand’s national universal health care scheme.

Thailand has one of the strongest and most equitable childhood immunization programs for its citizens in the region [3, 16], but gaps remain for ethnic minority [17] and migrant children [7, 13, 18, 19]. The national strategy is to vaccinate all infants against Hepatitis B at birth, and 2, 4, and 6 months of life, and vaccine coverage is reported at 97% [20]. For infants of mothers with chronic Hepatitis B infection, Hepatitis B immune globulin (HBIG) is also recommended but not always available. Myanmar’s expanded program of immunizations (EPI) gained strength under the civilian government from 2015 to 2020, including introduction vaccination against Hepatitis B at birth starting in 2016, which reached 17% of neonates by 2019. HBIG is largely unavailable in Myanmar due to cost. Completion of the three-dose Hepatitis B vaccine series at 2, 4, and 6 months was reported at 90% in 2019 [20], but vaccination disparities were significant between rural and urban children [21].

A recent retrospective review of vaccine utilization at Maharaj Nakorn Hospital, a tertiary government hospital in Chiang Mai, and clinics in Tak province run by the Shoklo Malaria Research Unit (SMRU) showed excellent coverage of birth dose Hepatitis B vaccine and HBIG. It also showed that birth vaccination in Chiang Mai was not different between Thai and non-Thai children [7]. Completion of the three Hepatitis B doses included in the EPI for migrants in Tak province has been documented at around 50% [7, 22]. Qualitative work from two studies in Tak province revealed that travel cost to the clinics, inability to miss work, and fear of arrest were key reasons for missed vaccination for children [13, 22]. No longitudinal work has followed migrant infants in these areas from birth to 6 months and beyond to prospectively assess hepatitis B vaccine completion, including vaccination outside the birth facility, and factors associated with missed and late doses.

**Fig. 1:**
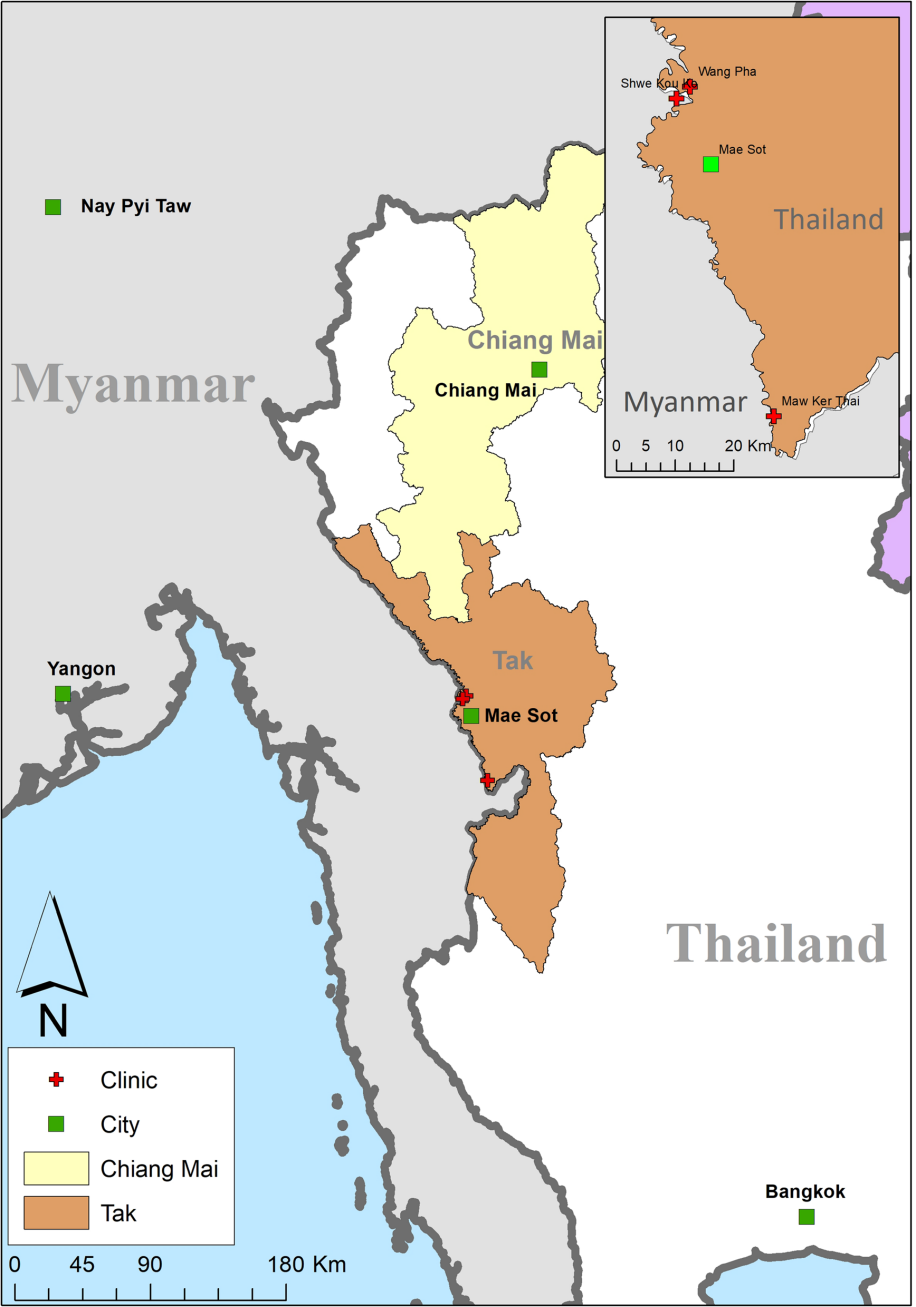
Map of study sites in Tak and Chiang Mai Provinces. Saraphee Hospital (SH) is located in the city of Chiang Mai. SMRU clinics are indicated by red crosses. Wang Pha and Maw Ker Thai clinics were included in this study. SMRU services opened at Shwe Kou Ko clinic in Myanmar following the COVID-19-related border closure in 2020 to reach patients who could not travel to Wang Pha. A free Myanmar Government vaccine clinic operated in Shwe Kou Ko at that time

In 2019 a series of knowledge, attitude and practice surveys were conducted at Sarapee hospital (SH) in Chiang Mai, a small hospital that serves the local migrant and Thai populations, and at SMRU clinics for migrant and minority communities in Tak province operated by SMRU (Fig. 1) [23]. Follow-up to determine the rate of vaccine completion was planned at each site, and happened to be disrupted by the COVID-19 pandemic. This analysis was undertaken to determine overall vaccine completion rates and estimate the impact of the pandemic and other socio-demographic factors on vaccine completion with the intention to improve vaccine programs for migrant workers in these and similar contexts.

## Methods

### Setting

The Shoklo Malaria Research Unit in Tak Province (Fig. 1) began pregnancy care in refugee camps in 1986, and in migrant communities in 1998. These free clinics offer contraception, antenatal, delivery [24, 25] and neonatal care [26], as well as general medical services. Since 2018 SMRU has worked with MFund [27], a non-profit insurance organization providing low-cost insurance for migrant workers in Thailand, covering emergency surgical services in the local Thai hospitals which are otherwise inaccessible to migrants due to cost.

Predominant languages spoken by clinic staff and patients are Burmese and Karen. Vaccines are provided to SMRU by the local government hospital to be administered free of charge to the migrant community, following the national vaccine schedule. Hepatitis B vaccination is given at delivery to infants of women with hepatitis B, and to all infants before discharge from the hospital. After infant vaccination, parents are given a vaccine card and the vaccine schedule is briefly explained along with the date for their next vaccination. Human resources for active follow-up of infants are not available.

SH is a government hospital in Chiang Mai that serves a large number of urban migrant workers particularly for physical examination, enrolment in health insurance, antenatal, delivery, newborn care, and vaccination. The vaccine clinic staff inform parents at each visit of the next scheduled vaccination and conduct reminder phone calls before appointments. The predominant language of the migrant population in Chiang Mai is Shan, which is related to but different from the Thai language. Vaccine services are provided in Thai and free of charge.

Additional vaccination services are provided free of charge at community public health facilities in Tak and Chiang Mai. Approximately half of the patients seeking care at the SMRU clinics at the time of this study lived in Myanmar and crossed the border temporarily from nearby villages for care in Thailand. This cross-border care-seeking was interrupted by the tightening of Thailand’s land borders due to the threat of COVID-19, with restrictions imposed starting in March 2020. Free vaccine clinics were also available in some communities in Myanmar at the time of this study.

### Survey

Briefly, a survey of knowledge, attitudes and practice (Additional file 1) was adapted from the literature [28] and administered to women in the first few days postpartum. The survey process and results of all women who participated are reported in detail elsewhere [23].

### Vaccine follow up

Approximately 1 year after birth, study files were reviewed for hepatitis B vaccine completion. Where vaccinations were incomplete, vaccine logbooks at the sites were reviewed to confirm vaccination status.

At SMRU a list was generated of all infants who were late or missing for vaccination. Attempts were first made to contact parents by phone. When this failed, home visits were attempted wherever possible. When contact could be made, study staff asked if infants had received vaccination elsewhere and if vaccines were missing or late, what the primary reason was.

At SH, in addition to the routine reminder call, hospital staff called parents who were over a month late for vaccination and ask about their reason for the missed vaccine. The research team further called parents or vaccine clinics to confirm reports of vaccines received elsewhere. At both SH and SMRU, paper documents of vaccination elsewhere were reviewed wherever possible.

### Statistical analysis

Statistical analysis was done using STATA version 15 (StataCorp, College Station, TX, USA) statistical software package. Categorical variables were summarized using proportions and crude association with the outcome variable was assessed using Chi-squared tests. Continuous variables were non-normal and summarized with medians and associations assessed using Mann-Whitney *U* test. Logistic regression was used for the multivariable model, with all variables included unless collinearity with a variable that was more strongly associated with the outcome prevented inclusion.

### Sample size and sampling approach

The proportion of SMRU infants completing vaccination was estimated at 0.5 based on previous work [7], while the SH proportion was expected to be higher and estimated at 0.7. Expecting a 4:1 proportion of participants at SMRU:SH based on the patient load at the respective facilities, a sample size of 57 participants at SH and 226 participants at SMRU would provide 80% power with an alpha of 0.05.

Sampling was cross-sectional: all women who delivered at the study sites during the sampling period were approached about the study and included in the survey if they provided consent.

## Results

Between September 10, 2019 and March 30, 2019, 253 women were surveyed, 200 from SMRU and 53 from SH. Results of this survey have been previously documented in detail [23]. Briefly, age, number of children, and Hepatitis B positivity were not significantly different between the sites (Table 1). SMRU participants were evenly split between Burman (93/200, 46.5%) and Karen (89/200, 44.5%) ethnicities, predominantly were undocumented (180/200, 90%), had non-government insurance (182/199, 91.5%), and primary school education (115/199, 57.8%). SH participants were predominantly of the Shan ethnicity (47/53, 88.7%), were documented (48/53, 90.6%), had government-issued insurance (40/53, 75.5%), and had no formal education (32/51, 62.7%). The median length of time staying at the participant’s current residence was longer at SH than SMRU.

**Table 1:** Demographics of the post-partum women participating in follow-up vaccination

Variable	Total (***N*** = 253)	SMRU (***n*** = 200)	Sarapee Hospital (***n*** = 53)	***P***-value
Age in years, median [IQR]	24 [21–29]	24 [20–29]	25 [22–32]	0.10^‡^
Ethnicity, n (%)				**< 0.001**^**†**^
Burman	94 (37.2)	93 (46.5)	1 (1.9)	
Shan	58 (22.9)	11 (5.5)	47 (88.7)	
Karen	90 (35.6)	90 (45.0)	0 (0)	
Other	11 (4.4)	6 (3.0)	5 (9.4)	
Health care coverage, n (%)				**< 0.001**^**†**^
Government insurance	46 (18.2)	6 (3.0)	40 (75.5)	
Private or NGO insurance	189 (74.7)	183 (91.5)	6 (11.3)	
No insurance	18 (7.1)	11 (5.5)	7 (13.2)	
Documentation status				**< 0.001**^**†**^
Documented	68 (26.88)	20 (10)	48 (90.57)	
Undocumented	185 (73.12)	180 (90)	5 (9.43)	
Length of time at this address years, median [IQR]	5.6 [2–13]	5 (1.6–13)	7 (3–15)	**0.04**^‡^
Number of children, n (%)				0.97^**†**^
1	120 (47.4)	95 (47.5)	25 (47.2)	
≥ 2	133 (52.6)	105 (52.5)	28 (52.8)	
Education, n (%)				**< 0.001**^**†**^
Not attend	72 (28.6)	40 (20.0)	32 (62.5)	
Elementary school	125 (49.6)	115 (57.5)	10 (19.2)	
Secondary school	55 (21.8)	45 (22.5)	10 (19.2)	
HBV status: Positive, n (%)	14 (5.6)	10 (5.0)	4 (7.8)	0.43^**†**^

Note: *SMRU* Shoklo Malaria Research Unit migrant clinics, *HBV*, Hepatitis B virus, *NGO* Non-governmental organization. ^‡^*P*-value from Mann-Whitney U test, ^†^*P*-value from chi-square test

Overall, knowledge about hepatitis B was higher for SMRU participants than SH participants (completely correct responses 46/200, 23.0% for SMRU vs 4/53, 7.6% for SH), but intention to vaccinate was similar (Table 2).

**Table 2:** Knowledge, attitudes, practice related to Hepatitis B vaccination

Variable	Total (***N*** = 253)	SMRU (***n*** = 200)	SH (***n*** = 53)	***P***-value
**All Knowledge answers correct**, n (%)	50 (19.8)	46 (23.0)	4 (7.6)	**0.01**^**†**^
**knowledge component of KAP survey results**, n (%)				**< 0.001**^**†**^
0–3 out of 7 answers correct	60 (23.7)	34 (17.0)	26 (49.1)	
4–5 out of 7 answers correct	75 (29.6)	61 (30.5)	14 (26.4)	
6–7 out of 7 answers correct	118 (46.6)	105 (52.5)	13 (24.5)	
**Attitude and practice components of KAP survey questions**, n (%)				
Are you willing to vaccinate your baby for HBV?	248 (98.0)	195 (97.5)	53 (100)	0.25^**†**^

Note: *SMRU* Shoklo Malaria Research Unit migrant clinics, *SH* Saraphi Hospital, *HBV* Hepatitis B virus, ^†^*P*-value from chi-square test

Novel data in this analysis focused on the outcome of vaccine completion. Despite the higher apparent knowledge about hepatitis B after counselling at SMRU, crude analysis **(**Table 3**)** showed that SMRU women had only 94/199 (47.2%) vaccine completion whereas participants from SH had a 46/53 (86.8%) vaccine completion. Ethnicity and documentation status were co-linear with site, with infants of documented migrants more vaccinated than undocumented, and Shan significantly more vaccinated than other groups. Older mothers were more likely to complete the vaccine series than younger mothers, and insured mothers fully vaccinated their children twice as often as uninsured mothers (57.7% vs. 27.8%).

**Table 3:** Crude and adjusted associations between key variables and completion of birth dose and three EPI doses of hepatitis B vaccine

Data		*n* (%)	***P***-value	aOR (95% CI)	*P*-value
Knowledge Questions All Correct	Yes	35/50 (70)	**0.02**	2.6 (1.2–5.6)	**0.014**
	No	105/203 (51.7)		*reference*	
Willing to vaccinate the baby for HBV	Yes	138/248 (55.7)	0.49	1.2 (0.2–7.8)	0.827
	No	2/5 (40)		*reference*	
Site	SH	46/53 (86.8)	**< 0.001**	14.3 (4.8–42.4)	**< 0.001**
	SMRU	94/200 (47)		*reference*	
Age	25–47	81/126 (64.3)	**0.004**	1.7 (1.0–3.1)	0.059
	16–24	59/127 (46.5)		*reference*	
Health care coverage	Insured	135/235 (57.5)	**0.02**	6.8 (1.4–34.5)	**0.020**
	Uninsured	5/18 (27.8)		*reference*	
Documentation status	Documented	47/68 (69.1)	**0.008**	–	
	Undocumented	93/185 (50.3)		–	
Length of time at current residence	≥ 6 months	136/236 (57.63	**0.006**	3.7 (1.0–13.5)	**0.045**
	< 6 months	4/17 (23.53)		*reference*	
Education	Not attend	43/72 (59.7)	0.12	*reference*	0.324
	Elementary school	61/125 (48.8)		1.5 (0.7–3.2)	
	Secondary school	35/55 (63.6)		2.0 (0.8–4.8)	
HBV status	Positive	10/14 (71.4)	0.21	1.4 (0.4–5.2)	0.644
	Negative	129/237 (54.4)		*reference*	
Ethnicity	Burman	44/94 (46.8)	**0.001**	**–**	
	Shan	46/58 (79.3)		–	
	Karen	45/90 (50)		–	
	Other	5/11 (45.5)		–	

Note: *HBV* Hepatitis B virus, *aOR* Adjusted odds ratio, *SMRU* Shoklo Malaria Research Unit migrant clinics, *SH* Sarapee Hospital

Willingness to vaccinate was previously reported to be unrelated to knowledge about hepatitis B [23]. In this dataset, there was no association between willingness to vaccinate and actual vaccine completion, which was not explored previously. Education level and hepatitis B status were also not significantly associated with vaccination.

On multivariable analysis **(**Table 3**)**, higher knowledge about hepatitis B, study site at SH, health care coverage and length of stay at current residence remained significantly associated with vaccine completion. Ethnicity and documentation were omitted because of high co-linearity with site and absence of association with vaccine completion within each site. The study site had the most dramatic association with vaccine completion with women surveyed at SH 14 times more likely to complete the vaccine series than those surveyed at SMRU. Having health insurance was associated with an almost 7 fold increase in the odds of completing the vaccine series, and duration of residence greater than 6 months almost quadrupled odds of vaccine completion. Though there was a trend in increasing vaccination with higher education and older age, this was not statistically significant, and there was likewise no evidence of an association between personal hepatitis B status and vaccine completion.

**Fig. 2:**
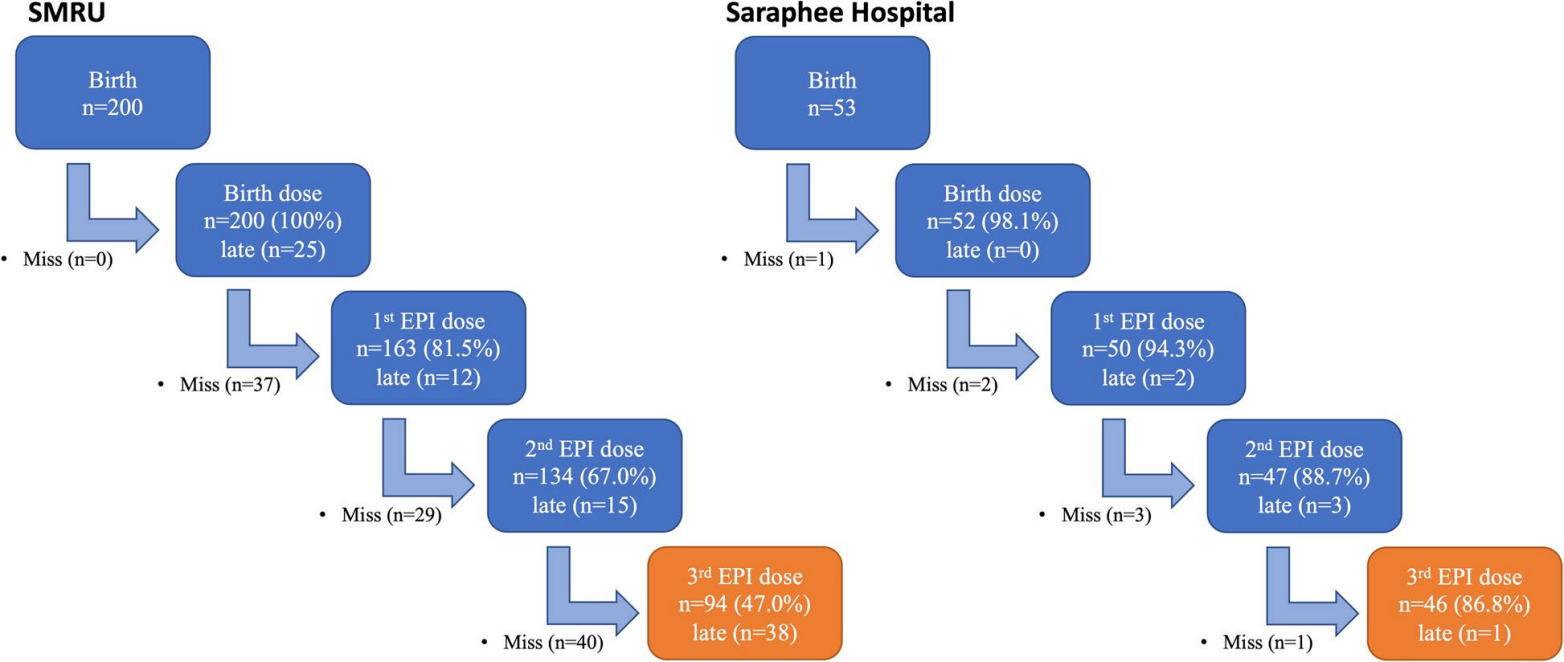
The Cascade of following-up vaccination at SMRU and SH. Abbreviations: EPI, Expanded program of immunizations

Overall there was steady loss to follow-up with each successive vaccine appointment (Fig. 2), most dramatically seen at SMRU clinics. Active follow up was attempted for 158/200 (79%) participants at SMRU due to missed or late vaccine appointments. Study staff were able to contact 31/158 (20%) by phone, and an additional 34/158 (21.5%) by home visit. At each time point, about 60% of missing participants could not be contacted and the reason for their lateness or loss to follow-up could not be determined.

Vaccination primarily occurred at the birth clinic (Supplementary Fig. S1, dark blue and green) but vaccination elsewhere increased over time. Among SH participants all vaccinations done outside SH were done through the Thai Health system (light green). All SMRU participants who were vaccinated outside of SMRU were vaccinated in Myanmar (light blue), and there was a sudden increase in vaccinations in Myanmar at the 6-month vaccine visit which largely fell after the border closure and travel restrictions for migrants at the end of March.

The reasons for missed appointments could finally only be determined for a small minority of infants, mainly due to inability to contact SMRU parents. Among those who could be contacted, onward migration (purple) was a common reason for missed vaccination at both sites but decreased by 6 months. Movement restriction due to COVID (yellow) was possible to document as a significant reason for missed vaccines at SMRU for the 4-month and 6-month visits.

**Fig. 3:**
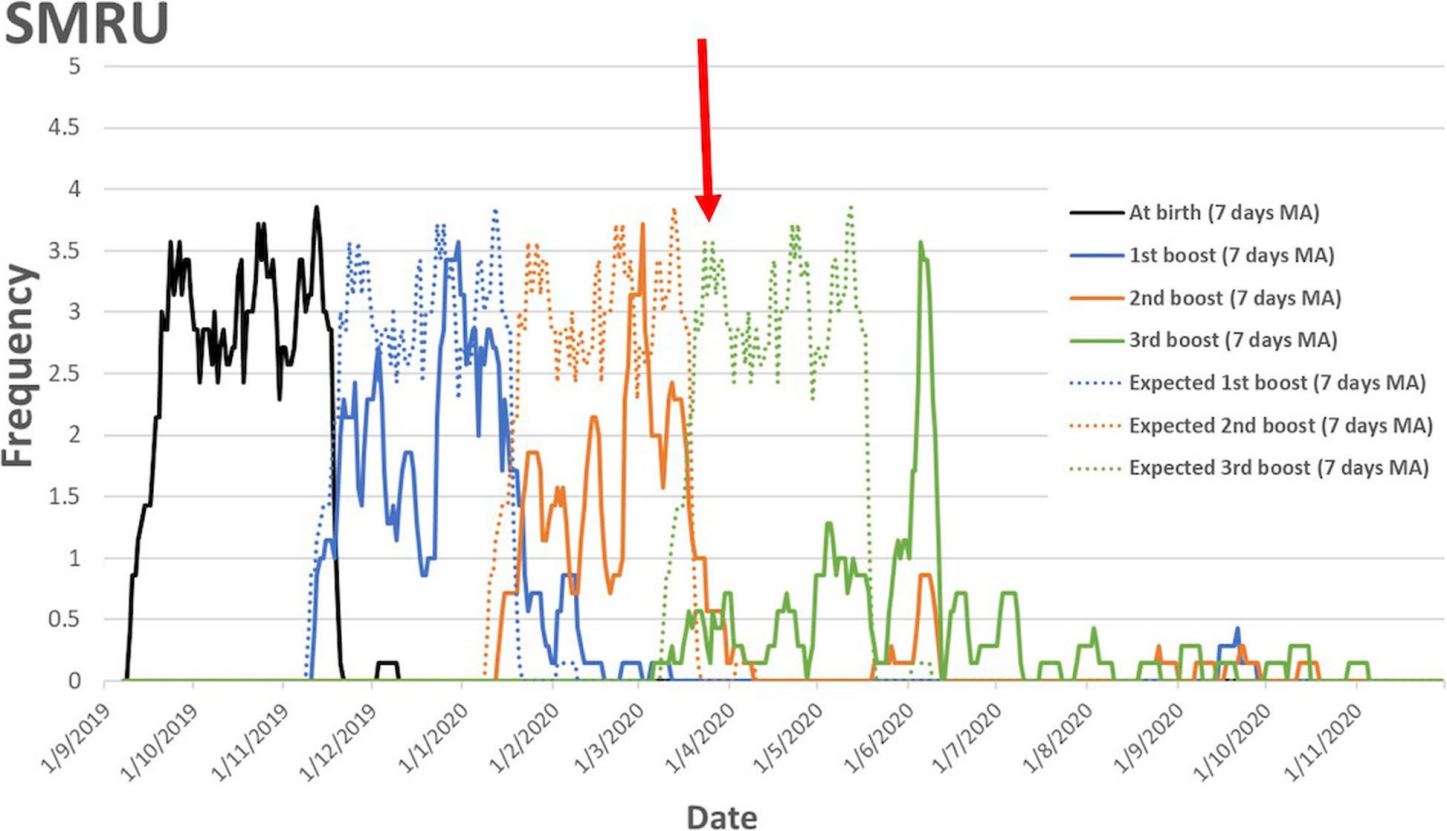
Timeline of expected (dotted line) and completed (solid line) vaccination at SMRU. The red arrow indicates the date of initiation of travel restrictions and border closure. Abbreviations: MA moving average

In order to visualize the impact of the pandemic-related travel restrictions on vaccine completion, expected (dotted line) and actual (solid line) vaccine visits were plotted over time for SMRU (Fig. 3). Timeliness and completion of vaccine visits dropped off abruptly after the travel restrictions which began 29th of March, 2020. There was a noticeable rebound in vaccination in June and July when restrictions were eased significantly.

At SMRU, by the end of the study, the vaccination status of 65/200 (32.5%) of infants was unknown (grey), resulting in considerable uncertainty about vaccine completion rate and reason for missed and late doses. Inability to contact participants at SH was much less common, and only 3/53 (5.7%) infants had unknown outcomes at the end of the study.

## Discussion

Hepatitis B infection is a major cause of death and disability worldwide, especially in areas of poor infant vaccine coverage. This study was initiated to better quantify hepatitis B vaccine completion among migrants with high rates of hepatitis B seropositivity in two different contexts in Thailand, to assess the impact of the COVID-19 pandemic on vaccination, and identify factors associated with improved vaccine completion. Strict travel restrictions imposed on migrants during the first and second quarters of 2020 hindered vaccination in Tak Province, but the impact of the pandemic was dwarfed by dramatic differences between the two study sites. Pervasive socio-economic marginalization of migrants in Tak province (indicated by low wages, under-documentation, unstable residence, and lack of government insurance) appears to be the main driver of vaccine non-completion in this population.

By far the most important factor determining vaccine completion was entering the study at SH in Chiang Mai versus SMRU clinics in Tak province. The SH experience, with vaccination completion of 86%, shows that it is possible to achieve high infant vaccination rates in migrant communities in Thailand, and suggests that the 90% goal could be attainable in some settings. However, at 47%, the vaccine completion at SMRU fell far below the target [4]. This difference could theoretically be due to three variables: individual factors of the parents, facility-level factors of the services, or broader socio-economic and political factors of the different settings. Though it is impossible to control for all individual-level factors, it is unlikely that this dramatic difference can be explained by demographics: women at SMRU were slightly more educated than women at SH, and age, parity, and hepatitis B seropositivity were similar. Predominant ethnic groups differed between sites but, as women delivering at SMRU had demonstrated *higher* understanding of hepatitis B and reported similar intention to vaccinate, cultural differences are not likely to account for the disparity. Residence less than 6 months at the current address was more common at SMRU and this has been associated with other negative health outcomes in this community [29], but the number of affected individuals was small.

There are differences between the clinical services at the sites. Unlike SMRU, SH does active follow-up for vaccine visits using phone calls. On the other hand, SMRU provides services in the preferred language of the migrant workers, which might be expected to improve follow up. Reported satisfaction with health services was similar in recent focus group discussions about choice of birth facilities in these same migrant populations [30], and vaccine services at both locations are free of charge.

Considering the relatively small differences between the individual characteristics and service provision, explanation of this fourteen-fold difference in vaccine completion requires consideration of broader socio-economic differences between the study sites. Social scientists have documented social and legal structures in Tak province which perpetuate a workforce of inadequately documented migrants who are then paid far below minimum wage and risk arrest when they travel outside their workplace [14, 15]. The pattern of documentation in this cohort supports this conclusion, with 90% of women at SH holding immigration documents compared with only 10% of women at SMRU. However, vaccine completion was only 30% among documented women at SMRU, reflecting the phenomenon in Tak Province by which migrants are marginalized and fear exploitation by authorities regardless of their documentation status as suggested by Reddy et al. [15]. The fact that no parents of infants born at SMRU reported vaccination in the free Thai government community vaccination clinics suggests a perceived lack of access to government services regardless of cost and official availability.

These apparent socio-economic barriers are consistent with qualitative studies [13, 22] in this population where major reasons for missed appointments included inability to miss work for even a day to get a child vaccinated, cost of travel, and fear of being arrested or fined on the way to the clinic. Distance to the facility increases both risk of arrest and the financial burden of travel, which often amounts to more than a day’s wages in Tak Province. Distance is generally shorter for urban (i.e. SH) than rural (i.e. SMRU) workers, and distance to services has been shown to have a negative impact on health service utilization among women seeking pregnancy services at SMRU [31]. Some programs in Tak province have shown potential to overcome these barriers such as school-based vaccination [18], and vaccine outreach programs run by district hospitals.

Other factors significantly associated with missed vaccines were lack of insurance, short duration of residence, and poor knowledge about hepatitis B after counselling. Young age had borderline significance. Taken together, these factors shed light on counselling opportunities that may improve vaccine follow-up. Targeting younger and more mobile mothers with extra encouragement to vaccinate, addressing barriers to insurance uptake, and adjusting the content of antenatal counselling to maximize understanding all have the potential to improve vaccine completion. As previously reported, almost all women intended to vaccinate their children, but higher knowledge did improve actual vaccination, after controlling for other factors. Previous work has shown that whenever possible, counselling strategies should be done in the parent’s preferred language, using non-technical terms, and should avoid overloading parents with too much information [23]. Approaches that optimize adult learning should be utilized as much as possible.

The magnitude of the impact of the COVID-19 pandemic on vaccination completion is difficult to estimate. Completed vaccination at SMRU clinics was less than 30%, compared with 50% in the previous studies [7, 22] but overall vaccination completion in the SMRU cohort almost reached 50% when doses received in Myanmar were included. However, there were substantial delays for vaccinations that occurred after the border closed. Since 60% of parents whose children were missing from vaccination could not be contacted, it is possible that a high proportion of these missed doses were due to COVID-19. The shift in reasons for missed visits from onward mobility to lack of mobility (due to pandemic travel restrictions) suggests a scenario where these marginalized children are chronically left behind – whether by the need of their parents to migrate for income or by the restrictions that limit both mobility and livelihoods during the pandemic. The impact of the border closure was mitigated by the availability of Myanmar vaccination services accessed by 38 infants receiving 56 hepatitis B doses in Myanmar, collectively. These services have now largely collapsed following the 2021 *coup d’etat* so the current degree to which vaccination has been disrupted is likely significantly greater.

There are several limitations to this analysis. Designed to identify classic demographic factors typically used in epidemiological studies, this analysis stumbled into socio-economic and political territory that it was not designed to elucidate. However, the available data on education, knowledge, documentation and insurance status corroborated phenomena described in more detail in the social science literature. The adjusted OR for several of the independent variables had wide confidence intervals reflecting the small number of participants in some groups. Determining the reason for loss to follow up is a chronic challenge that was not overcome in this work, where a reason for loss to follow up could not be determined in 60% of cases. This challenge highlights the difficulty of using mobile-phone-based interventions in the Tak migrant population where only 20% of participants could be contacted by phone. This contrasts with earlier findings that suggested cell phones were potentially helpful for childhood immunization programs in the Thai border population [32]. Finally, the study may have accessed different types of migrants at each study location. There are some documented migrants in Tak province who do seek care at the local Thai hospitals and may resemble the population seeking care at SH more than the children vaccinated at SMRU. Likewise, there are undocumented migrants in Chiang Mai who may not be seeking birth or vaccination services in the government system, and they were not represented in this study.

Case studies of migration and health are inevitably grounded in a specific geographic context. However, the scenario of migrant workers from an unstable state existing with various levels of documentation and acceptance within host countries with stronger economies and health systems is a global phenomenon of urgent importance [9–11]. Lessons from this setting are likely to be applicable elsewhere, though similar research in other settings would be valuable. As the world still struggles to recover from wave after wave of COVID-19 infections driven largely by unvaccinated communities, determinants of vaccination uptake in marginalized populations are particularly relevant.

Despite our best efforts to trace patients, this study was only partially able to quantify vaccine completion rates for migrants and elucidate reasons for missed doses. It is apparent that good vaccination completion rates are possible in some migrant populations, but fall short of vaccination rates among Thais. Barriers remain significant in rural border communities. Improvements in vaccination rates may be achievable through improved antenatal care education and targeting interventions for young mothers and those without insurance. Active follow up would likely be beneficial, but requires funding and personnel and should not rely heavily on phone contact. Interventions that decrease the cost of vaccination for migrant workers by decreasing travel distance, risk of arrest, and lost time from work such as community-level vaccine outreach or school-based vaccine programs are likely to improve uptake. Overall, the data from this cohort suggest that by far the greatest gains in vaccination coverage can be expected by strengthening mechanisms to include all migrant workers in national health systems.

## Conclusions

 Achievement of high levels of hepatitis B vaccination in migrant communities is important and feasible, and requires inclusive policies that integrate migrants into national health and social services. This is more urgent than ever during the COVID-19 era. Further work should prospectively measure the effect of streamlined documentation processes and expanded insurance coverage in border migrant settings on vaccine coverage and disease prevention. Effective adult learning strategies should be used in antenatal care counselling, and counselling content should focus on the most meaningful information to improve knowledge uptake and subsequent vaccination by parents. Specific efforts to reach the most vulnerable may involve community- or school-based vaccination to reduce cost and security barriers, and programs should not rely on cell-phone-based strategies only.

## Supplementary Information

**Additional file 1.**

**Additional file 2:Supplementary Fig. S1.** Vaccination outcomes and reason for missed vaccinations by expected vaccine timepoint.

## Data Availability

The datasets generated and/or analysed during the current study are not publicly available due to the sensitive nature of the data and the vulnerable population under study, but are available from the corresponding author on reasonable request.

## Abbreviations

EPI: Expanded program of immunizations
HBIG: Hepatitis B immune globulin
HBV: Hepatitis B virus
NGO: Non-governmental organization
SH: Saraphee Hospital
SMRU: Shoklo Malaria Research Unit
