# Supplementary material for: Who is protected? Determinants of hepatitis B infant vaccination completion among a prospective cohort of migrant workers in Thailand during the COVID-19 pandemic
Source: Int J Equity Health. 2022 Dec 30;21:190. doi: 10.1186/s12939-022-01802-5 (PMC9803398; doi:10.1186/s12939-022-01802-5)
Supplement: Supplementary file 1 — Additional file 1. [file 12939_2022_1802_MOESM1_ESM.docx]

# APPENDIX. KAP Survey questions in English

KAP survey with three components: Sociodemographic information; HBV awareness and knowledge; Attitudes toward HBV.

Sociodemographic questions to be considered:

1. Age
2. Length of residency at current residence
3. How many children do you have?
4. Highest level of education

HBV awareness and knowledge (answers “yes”, “no”, “don’t know”):

1. HBV can be transmitted through blood transfusion.
2. HBV can be transmitted through unprotected sexual intercourse.
3. HBV can be transmitted from mother to fetus.
4. HBV can be transmitted through the unsafe use of needles or sharps.
5. HBV can cause damage to the liver.
6. A person can be infected with HBV and not show any signs.
7. There is a vaccine for HBV.

HBV attitudes (answers “yes” “no” “don’t know”)

1. Did you get a screening test for HBV? *[NB not for partner]*
2. Did you feel forced to get the test done?
3. Are you willing to get your baby the vaccine for HBV?
4. I told my spouse my HBV status.
