# Supplementary figures and images for: Who is protected? Determinants of hepatitis B infant vaccination completion among a prospective cohort of migrant workers in Thailand during the COVID-19 pandemic
Source: Int J Equity Health. 2022 Dec 30;21:190. doi: 10.1186/s12939-022-01802-5 (PMC9803398; doi:10.1186/s12939-022-01802-5)

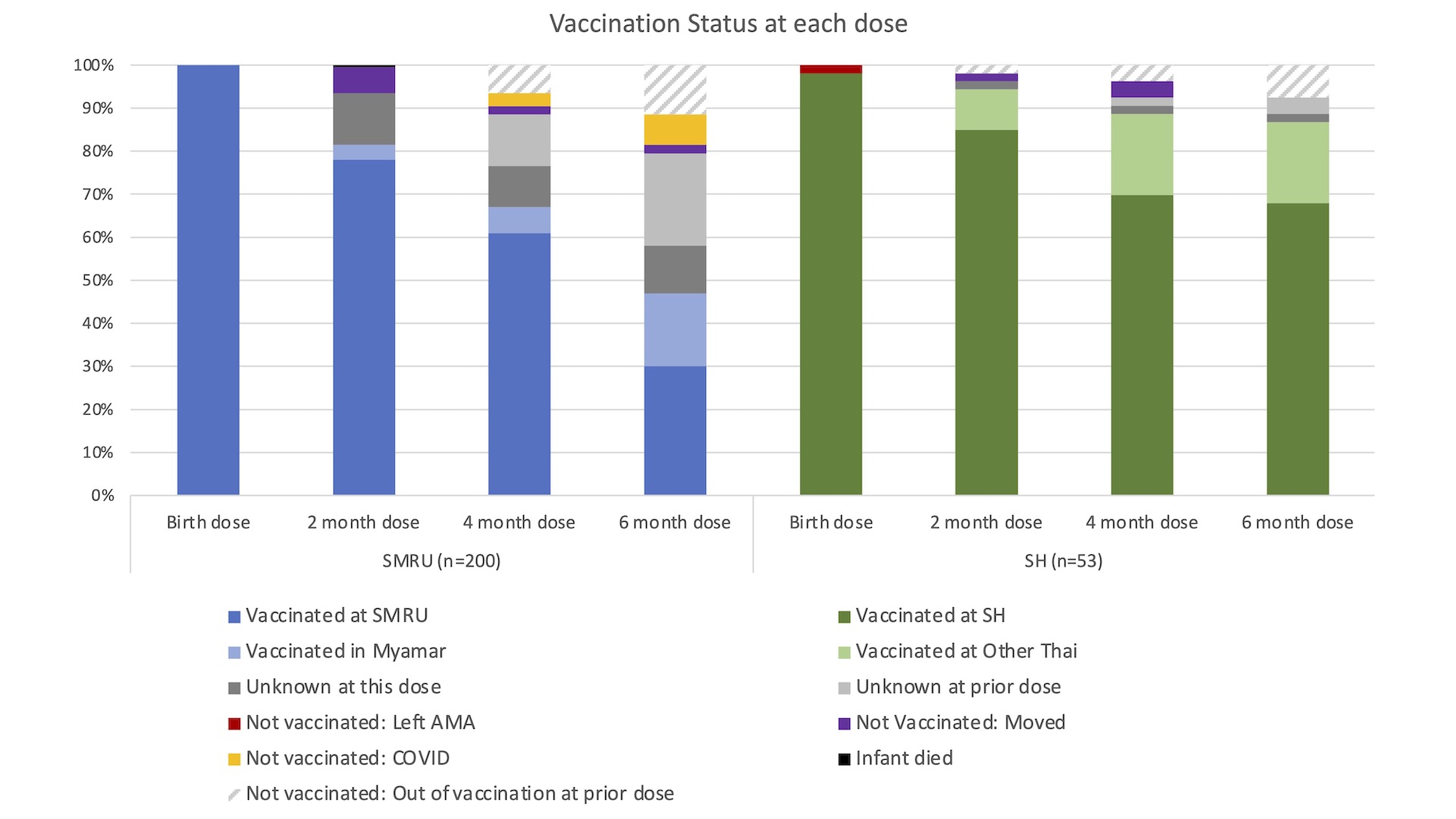

Supplement: Supplementary file 2 — Additional file 2:Supplementary Fig. S1. Vaccination outcomes and reason for missed vaccinations by expected vaccine timepoint. [file 12939_2022_1802_MOESM2_ESM.jpg]
